# Correlation Between Ictal Signs and Anatomical Subgroups in Temporal Lobe Seizures: A Stereoelectroencephalography Study

**DOI:** 10.3389/fneur.2022.917079

**Published:** 2022-06-10

**Authors:** Bo Zhang, Jing Wang, Mengyang Wang, Xiongfei Wang, Yuguang Guan, Zhao Liu, Yao Zhang, Changqing Liu, Meng Zhao, Pandeng Xie, Mingwang Zhu, Tianfu Li, Guoming Luan, Jian Zhou

**Affiliations:** ^1^Department of Neurosurgery, Center of Epilepsy, Beijing Institute for Brain Disorders, Beijing Key Laboratory of Epilepsy Research, Sanbo Brain Hospital, Capital Medical University, Beijing, China; ^2^Department of Neurology, Center of Epilepsy, Beijing Institute for Brain Disorders, Sanbo Brain Hospital, Capital Medical University, Beijing, China; ^3^Department of Radiology, Sanbo Brain Hospital, Capital Medical University, Beijing, China; ^4^Department of Brain Institute, Center of Epilepsy, Beijing Institute for Brain Disorders, Beijing Key Laboratory of Epilepsy Research, Sanbo Brain Hospital, Capital Medical University, Beijing, China

**Keywords:** temporal lobe epilepsy, epileptic seizures, ictal semiology, cluster analysis, stereoelectroencephalography

## Abstract

**Objective:**

Ictal semiology is a fundamental part of the presurgical evaluation of patients with temporal lobe epilepsy. We aimed to identify different anatomical and semiologic subgroups in temporal lobe seizures, and investigate the correlation between them.

**Methods:**

We enrolled 93 patients for whom stereoelectroencephalography exploration indicated that the seizure-onset zone was within the temporal lobe. Ictal signs and concomitant stereoelectroencephalography changes were carefully reviewed and quantified, and then cluster analysis and the Kendall correlation test were used to associate ictal signs with the temporal structures of patients.

**Results:**

Clustering analysis identified two main groups of temporal structures. Group 1 consisted of the medial temporal lobe structures and the temporal pole, which were divided into two subgroups. Group 1A included the hippocampal head, hippocampal body, and amygdala, and this subgroup correlated significantly with oroalimentary automatisms, feeling of fear, and epigastric auras. Group 1B included the hippocampal tail, temporal pole, and parahippocampal gyrus, and this subgroup correlated significantly with manual and oroalimentary automatisms. Group 2 consisted of the cortical structures of the temporal lobe and was also divided into two subgroups. Group 2A included the superior and middle temporal gyrus, correlated significantly with bilateral rictus/facial contraction, generalized tonic–clonic seizure, and manual automatisms. Group 2B included Heschl's gyrus, the inferior temporal gyrus, and the fusiform gyrus, and this subgroup correlated significantly with auditory auras, focal hypokinetics, unilateral upper and lower limbs tonic posture/clonic signs, head/eye deviation, unilateral versive signs, and generalized tonic–clonic seizure.

**Significance:**

The temporal structures can be categorized according to the level at which each structure participates in seizures, and different anatomical subgroups can be correlated with different ictal signs. Identifying specific semiologic features can help us localize the epileptogenic zone and thus develop stereoelectroencephalography electrode implantation and surgical resection protocols for patients with temporal lobe epilepsy.

## Introduction

Temporal lobe epilepsy (TLE) is the most common type of refractory focal epilepsy in adolescents and adults, and surgery is often an effective therapy for patients with TLE (1–3). Semiologic analysis is an essential method for localizing the epileptogenic zone (EZ) in presurgical evaluations. Some early works have attempted to distinguish TLE from other lobe epilepsies by analyzing semiologic features (4, 5). Subsequently, several studies attempted to make semiologic distinctions between left- and right-side TLE or between medial temporal lobe epilepsy (MTLE) and lateral temporal lobe epilepsy (LTLE) (6–9). However, as a network disorder, ictal signs of seizures are produced by dynamic discharges that can spread either close to or far from those origins. In other words, ictal signs are not entirely produced at the EZ, and some of those signs occur because the discharges have spread from the EZ to certain areas. To better understand the correlation between different semiologic patterns and anatomical structures of temporal lobe seizures, it is insufficient to divide patients into left and right or medial and lateral groups or limit evaluations to patients who have been surgically cured. Thus, it is necessary to refine the spatiotemporal evolution of this activity and its associated clinical signs and anatomical structures. Because of the intuitive and high-resolution features of stereoelectroencephalography (SEEG) (10, 11), a three-dimensional view of seizure dynamics is possible (12). Using this approach, when a particular ictal sign is produced, it can be easier to find a more specific intracranial discharge region associated with it.

Studies on semiology aimed to precisely localize the origin of seizure activity and guide the development of electrode implantation protocols for SEEG or surgical resection protocols for EZs. We deliberately selected patients whose SEEG examination clearly showed that the seizure-onset zone (SOZ) was entirely in the temporal lobe and classified the epileptic discharge regions according to the anatomical structures. Finally, we identified several anatomical and semiologic subgroups and correlated ictal signs with temporal structures by analyzing quantified ictal signs and SEEG changes, which can assist in the precise localization of the EZ to better guide the development of electrode implantation protocols for SEEG and surgical resection protocols for EZs.

## Methods

### Patient Selection

The authors collected patients with drug-resistant focal epilepsy who underwent SEEG at the epilepsy center in Sanbo Brain Hospital Capital Medical University from April 2012 to December 2019. Patients for whom SEEG exploration defined the SOZ as being within the temporal lobe were selected.

We included a total of 93 patients who met the following inclusion criteria: (1) The epileptic discharge recorded by SEEG started entirely in the temporal lobe, regardless of unilateral or bilateral and the range of conduction. (2) Neurological examination findings were normal, and no previous neurosurgical surgery was performed. (3) Patients did not have severe postoperative complications (cerebral infection, hemorrhage, etc.). We excluded patients whose eventual intracranial recording was inconclusive or in whom the SOZ involved extratemporal regions. This study was approved by the Ethics Committee of Sanbo Brain Hospital, Capital Medical University.

### Presurgical Evaluation

Prior to selection for SEEG, we performed a phase of thorough non-invasive presurgical evaluation. The data included detailed clinical history, neuropsychology assessment, computed tomography (CT), magnetic resonance imaging (MRI), surface video-electroencephalography (VEEG) recording, magnetoencephalography (MEG) and 18F-fluorodeoxyglucose positron emission tomography (PET). Each patient underwent CT scans, MRI scans, and VEEG recordings. Besides, MEG and PET were performed for those with ambiguous MRI and VEEG results.

For SEEG, the indications followed French guidelines (13, 14). Every trajectory blueprint of SEEG was designed by one neurologist and two neurosurgeons according to the results of the non-invasive tests. Intracerebral multiple contact electrodes were implanted under general anesthesia. Moreover, a robotic stereotactic assistant helped with the procedure. CT was performed 6 h postoperatively to confirm that there was no intracranial hemorrhage. The accuracy of the electrode implantation position was determined by CT-scan/pre-implant MRI data fusion. After implantation, all patients underwent a 128-channel VEEG recording to document the patient's habitual seizures. Once monitoring was completed, two neuroelectrophysiologists reviewed the recorded material, and any differences were then resolved by joint consensus.

### Analysis of Anatomic-Electroclinical Features

Each patient's seizure was analyzed following the method developed by a previous study of frontal lobe seizures (15). Reviewing all seizures recorded by SEEG, we counted the same anatomic-electroclinical patterns of one patient as one form of seizure and the different anatomic-electroclinical patterns of a patient as different forms of seizures. Moreover, present in different patients, even if the anatomic-electroclinical patterns were the same, we counted them as different forms of seizures. In total, 112 forms of seizures and 22 ictal signs recorded by SEEG were collated; semiologic categories and nomenclature methods have been described in previous studies (15–17). In addition, this study was conducted on the basis of SEEG. All of the ictal signs we recorded were appeared during SEEG monitoring. Due to the limited time of SEEG recording, the semiology does not appear every time, the seizures in some patients occur during sleep, and the patient forgets after seizures, although some auras are common semiologic features of temporal lobe epilepsy, they were recorded only once or not in our study, such as Déjà vu, dizziness, visual aura, and olfactory aura. Therefore, they were not included in this study. In addition, the verbal mostly occurs in seizures caused by EZs in the non-dominant hemisphere. In this study, a total of 6 patients presence verbal, of which 5 patients had EZs in the non-dominant hemisphere and only 1 patient had EZ in the dominant hemisphere. For each seizure form, the presence or absence of 22 ictal signs was noted. Furthermore, we established an overall semiologic score for each form of seizure, with values ranging from 0 (=absence of that sign) to a maximum of 2 for major features (=constant and early sign present in each seizure), with a score of minor features of 1 (=sign not always present).

We similarly analyzed a time window for the anatomical structures involved in each seizure, starting from electrical onset to the full emergence of all semiologic elements (15). The individual anatomical structure was scored based on the degree of involvement in the seizure (the earliness of the appearance of ictal activity in that structure and the extent of electrical activity change in frequency, amplitude, or rhythmicity compared to preictal activity in the same structure). A structure not involved in seizure activity (without any electroencephalography modification) was scored as 0. While a structure immediately involved in electrical onset or with low voltage rapid discharge that occurred before clinical semiology arose was scored as 2, the intermediate score 1 was used when seizure activity was observed later.

### Statistical Analysis

First, based on the two matrices (signs ^*^ seizure forms and structures ^*^ seizure forms), we computed two dissimilarity matrices, which were entered into hierarchical clustering analysis (SPSS, version 26.0). Cluster analysis allowed for the classification of the variables. Thus, ordered sequences of signs and structures were obtained and arranged according to the frequency of their co-occurrence, and a dendrogram of rank arrangement relations representing signs or structures was obtained. The proximity of the variables in the dendrogram represents the degree of similarity among them. That is, ictal signs closer to each other in the dendrogram are often present together during seizures, while structures closer to each other in the dendrogram are often concurrently involved during seizures. Finally, a Kendall correlation test was conducted to study the correlation between ictal signs and structures, and P < 0.05 indicated a significant correlation.

## Results

### General Characteristics

We included 93 patients (44 males, 49 females) with seizures arising from the temporal lobe. The mean age at recording was 26.1 ± 8.5 years; the mean epilepsy duration was 12.6 ± 8.0 years. Each patient was monitored by SEEG for 2 to 16 days, with an average duration of monitoring of 6.5 ± 2.6 days, and 1–11 (mean 4.7, median 4) seizures were recorded per patient. Four hundred and fourty one seizures were recorded with SEEG and classified into 112 seizure forms. In all recorded seizures, clinical semiology began after the onset of SEEG ictal discharge. The main ictal signs of 93 patients are shown in Table 1. To improve the accuracy, for the statistics of the epigastric auras, feeling of fear, and auditory auras, we combined the records in the clinical history. In total, we divided 17 anatomical structures (11 temporal structures and 6 extratemporal structures). The SEEG recordings for each anatomical structure in the 112 forms are shown in Table 2.

**Table 1:** The main ictal signs of 93 patients.

**Ictal sign**	**Number (%)**	**Ictal sign**	**Number (%)**
Int-gest-motor	18 (19)	Hyperkinetic	21 (23)
Uni-upper limb tonic/clonic signs	42 (45)	Verbal	6 (6)
Uni-facial tonic/clonic signs	11 (12)	Head/eye deviation	26 (28)
GTCS	30 (32)	Manual automatisms	55 (59)
Uni-tonic posture/clonic signs	10 (11)	Auditory auras	6 (6)
Staring/behavioral arrest	24 (26)	Autonomic seizure	37 (40)
Oroalimentary automatisms	53 (57)	Focal hypokinetics	22 (24)
Asymmetric tonic posture	7 (8)	Epigastric auras	13 (14)
Generalized hypokinetic	17 (18)	Feeling of fear	22 (24)
Bilateral rictus/facial contraction	5 (5)	Vocal	14 (15)
Bilateral upper-limb tonic posture	9 (10)	Unilateral versive signs	23 (25)

*int-gest-motor, integrated gestural motor behaviors; uni-upper limb tonic/clonic signs, unilateral upper limb tonic posture/clonic signs; uni-facial tonic/clonic signs, unilateral facial tonic/clonic signs; GTCS, generalized tonic–clonic seizure; uni-tonic posture/clonic signs, unilateral upper and lower limbs tonic posture/clonic signs*.

**Table 2:** The SEEG recordings for each anatomical structure in the 112 forms.

**Anatomical structure**	**With electrodes** **implantation** **(%)**	**With epileptic** **discharge** **(%)**	**Anatomical** **structure**	**With electrodes** **implantation** **(%)**	**With epileptic** **discharge** **(%)**
STG	99 (88)	36 (32)	HT	59 (53)	53 (47)
MTG	111 (99)	45 (40)	AMYG	80 (71)	67 (60)
ITG	72 (64)	14 (13)	F	71 (63)	30 (27)
FFG	40 (36)	10 (9)	I	99 (88)	59 (53)
HES	49 (44)	12 (11)	O	37 (33)	9 (8)
TP	69 (62)	50 (45)	P	57 (51)	21 (19)
PHG	78 (70)	56 (50)	AMCG	39 (35)	15 (13)
HH	105 (94)	102 (91)	PCG	78 (70)	36 (32)
HB	72 (64)	60 (54)			

*STG, superior temporal gyrus; MTG, middle temporal gyrus; ITG, inferior temporal gyrus; FFG, fusiform gyrus; HES, Heschl's gyrus; TP, temporal pole; PHG, parahippocampal gyrus; HH, hippocampal head; HB, hippocampal body; HT, hippocampal tail; AMYG, amygdala; F, frontal lobe; I, insula lobe; O, occipital lobe; P, parietal lobe; AMCG, anterior and middle cingulate gyrus; PCG, posterior cingulate gyrus*.

### Cluster Analysis and Anatomic-Electroclinical Correlations

**Figure 1:**
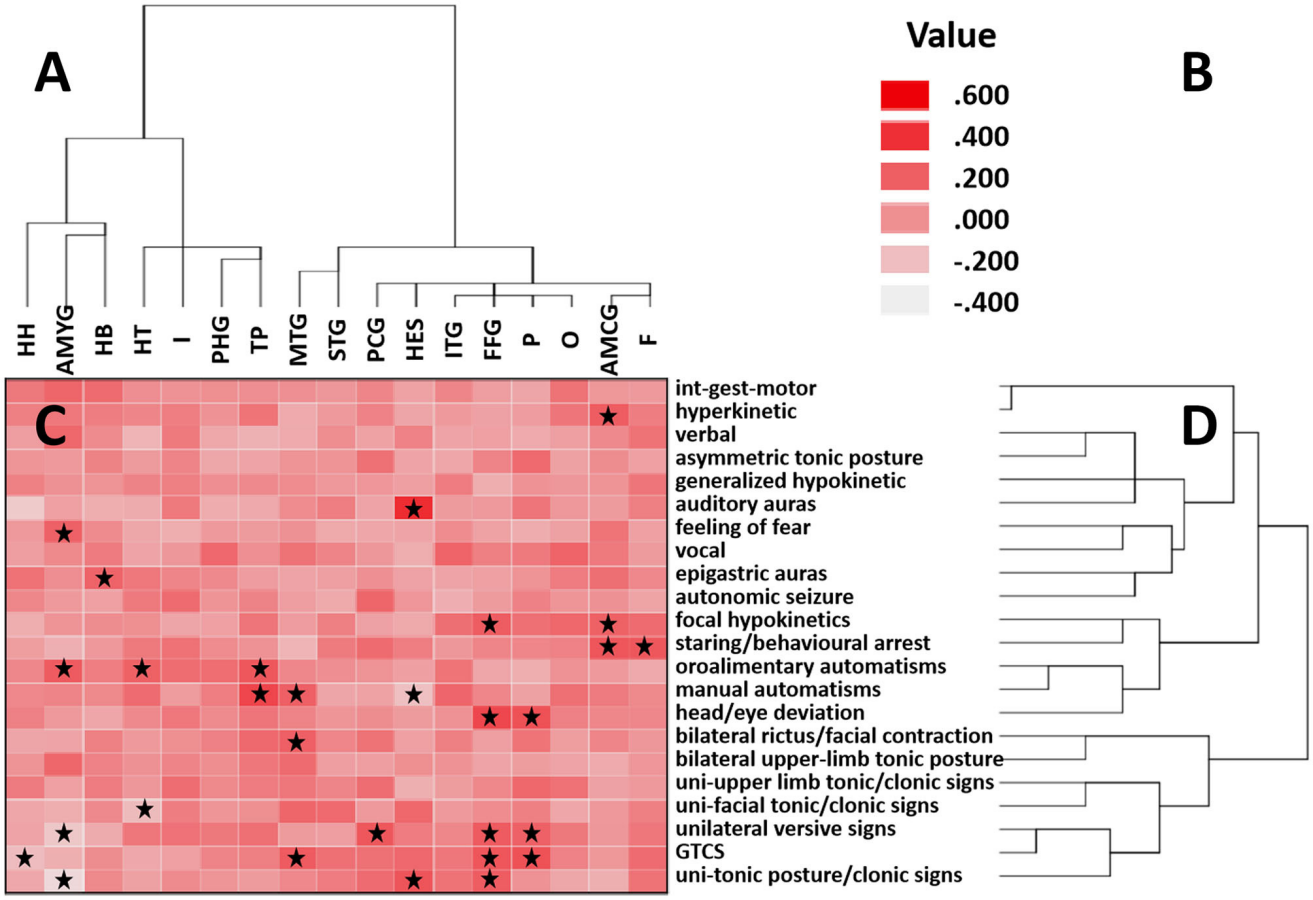
Correlation matrix between cortical areas and clinical features. **(A)** Clustering of brain regions is shown on the horizontal axis. **(B)** Heatmap color corresponds to the correlation coefficient. Positive values indicate a positive correlation, and negative values indicate a negative correlation. **(C)** Correlation between clinical features and brain regions is indicated by a correlation heatmap. The starred squares shown in the squares denote a significant Kendall correlation (*P* < 0.05). **(D)** Clustering of clinical signs is shown on the vertical axis. In these two ordered sequences, neighboring regions and neighboring signs occur more frequently together than distant sequences. HH, hippocampal head; AMYG, amygdala; HB, hippocampal body; HT, hippocampal tail; I, insula; PHG, parahippocampal gyrus; TP, temporal pole; MTG, middle temporal gyrus; STG, superior temporal gyrus; PCG, posterior cingulate gyrus; HES, Heschl's gyrus; ITG, inferior temporal gyrus; FFG, fusiform gyrus; P, parietal; O, occipital; AMCG, anterior and middle cingulate gyrus; F, frontal. int-gest-motor, integrated gestural motor behaviors; uni-upper limb tonic/clonic signs, unilateral upper limb tonic posture/clonic signs; uni-facial tonic/clonic signs, unilateral facial tonic/clonic signs; GTCS, generalized tonic–clonic seizure; uni-tonic posture/clonic signs, unilateral upper and lower limbs tonic posture/clonic signs.

The results of the hierarchical clustering analysis of ictal signs and temporal structures are shown in Figure 1. Ictal signs of the patients were clustered into two groups. One group consisted entirely of elementary motor signs. In comparison, the other group contained the remaining signs (Figure 1D), namely, automatisms, integrated gestural motor behavior, auras, autonomic changes, asymmetric tonic posture, head/eye deviation, etc. The temporal structures were clustered into two groups (Figure 1A). Group 1 consisted of medial temporal lobe structures and the temporal pole, and Group 2 consisted of cortical structures of the temporal lobe. Regarding the extratemporal lobe structures, the temporal structures of Group 1 were clustered with the insula lobe, indicating that the abnormal discharge of anatomical structures in Group 1 was transmitted more readily to the insula lobe, particularly in the hippocampal tail, temporal pole, and parahippocampal gyrus. The temporal structures of Group 2 were clustered with the frontal lobe, parietal lobe, occipital lobe, and cingulate gyrus, also indicating that the abnormal discharge of the anatomical structures in Group 2 was more readily transmitted to these areas, especially in the inferior temporal gyrus and the fusiform gyrus, with signals more readily transmitted to the occipital and parietal lobe.

Each of the groups can be subdivided into two subgroups. Structures within each subgroup are often closer in anatomical positions than structures that are not in the same subgroup. Group 1 can be divided into Groups 1A, B. Group 1A included the hippocampal head, hippocampal body, and amygdala. These areas were relatively inward in the medial temporal lobe. Furthermore, the amygdala correlated significantly with oroalimentary automatisms and feeling of fear. The hippocampal body had a significant correlation with epigastric auras. No clinical signs significantly correlated with the hippocampal head. Group 1B included the hippocampal tail, temporal pole, and parahippocampal gyrus. The hippocampal tail and parahippocampal gyrus are at relatively posterior and external sites in the medial temporal lobe, and the temporal pole is at a more lateral and anterior level. The hippocampal tail correlated significantly with oroalimentary automatisms. The temporal pole correlated significantly with manual and oroalimentary automatisms. No clinical signs significantly correlated with the parahippocampal gyrus.

**Figure 2:**
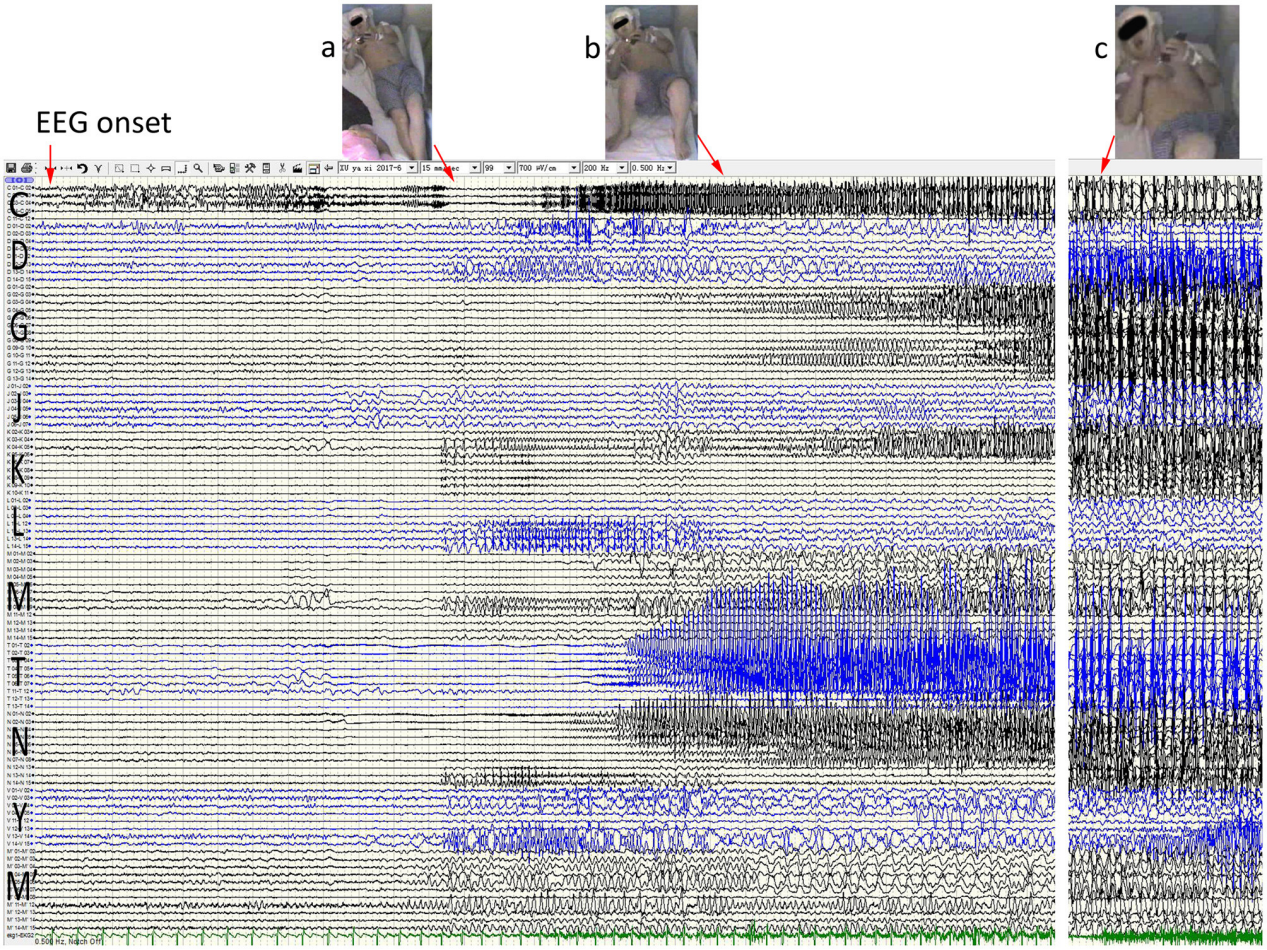
Anatomic-electroclinical features at onset stage of seizures in a patient. Ictal stereoelectroencephalography (SEEG) shows a continuous spike rhythm in the hippocampal body at electrical onset, transmits early to the temporal pole and the insula lobe, and then presents increased heart rate (a) and automatisms (b). With the spread of epileptic discharge, the extensive temporal and extratemporal cortex exhibited rhythmic spike-and-alow waves, followed by right upper-limb tonic posture and right rictus contraction (c).

**Figure 3:**
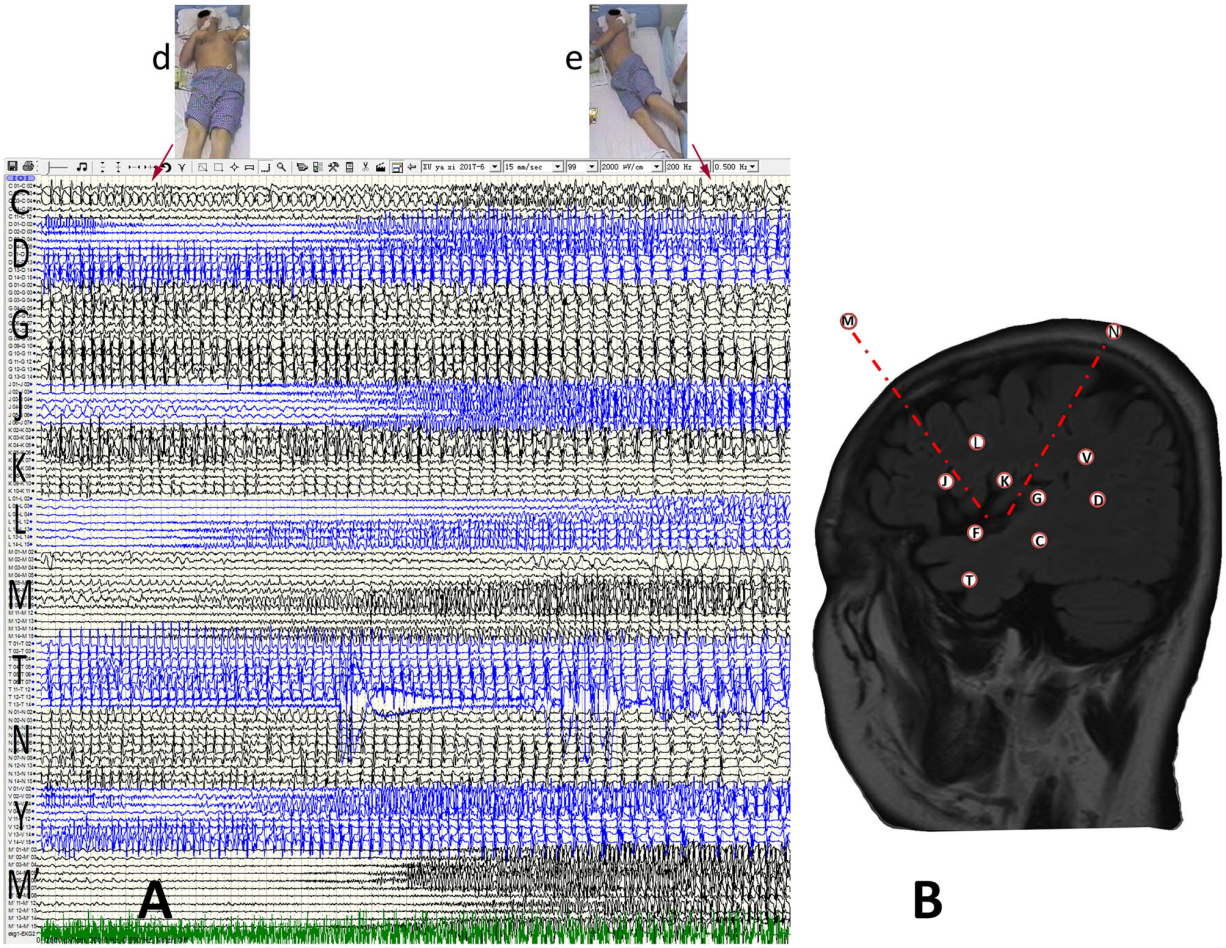
Anatomic-electroclinical features at final stage of seizures in a patient. **(A)** The patient developed bilateral upper-limb tonic posture (d) with dystonia after continuing to spread to the contralateral cerebral cortex, and followed by GTCS (e). **(B)** The SEEG design protocol of the patient.

Group 2 can also be divided into two subgroups, Groups 2A, B. Group 2A included the superior and middle temporal gyrus, located in relatively above positions of the temporal cortex. The middle temporal gyrus was significantly correlated with bilateral rictus/facial contraction, generalized tonic–clonic seizure (GTCS), and manual automatisms. No clinical signs were significantly correlated with the superior temporal gyrus. Group 2B included Heschl's gyrus, the inferior temporal gyrus and the fusiform gyrus. Heschl's gyrus is located in the lower wall of the lateral sulcus, and the other two structures are located in relatively low positions of the temporal cortex. Heschl's gyrus correlated significantly with auditory auras and unilateral upper and limbs tonic posture/clonic signs. The fusiform gyrus correlated significantly with focal hypokinetics, unilateral upper and lower limbs tonic posture/clonic signs, head/eye deviation, unilateral versive signs, and GTCS. No semiology correlated significantly with the inferior temporal gyrus. Figures 2, 3 describe the anatomic-electroclinical features at different stages of seizure in the same patient.

## Discussion

Luders (18) divided seizure-related brain regions into five zones: the symptomatogenic zone, irritative zone, seizure-onset zone, epileptogenic lesion, and functional deficit zone. Their scopes often did not coincide with each other. To better understand the correlation between different semiologic patterns and brain regions, it is necessary to refine the spatiotemporal evolution and correlated semiologic features of this activity. By reporting the anatomic-electroclinical features of 93 patients with TLE, we divided temporal structures into two groups based on the degree of involvement in seizures by cluster analysis. Structures within the same group are generally closer in their anatomical positions. Moreover, we found some significant correlations between several ictal signs and the temporal structures of each group. These structures may be the symptomatogenic zone of correlated signs or important in the related epileptic network.

### The Anatomic-Electroclinical Correlation of Group 1

Group 1 was composed of medial temporal lobe structures and the temporal pole, which correlated significantly with feeling of fear, epigastric auras and automatisms. The grouping results indicate that the medial temporal lobe structures and the temporal pole are often jointly involved during seizures. The temporal pole plays an important role in the occurrence and transmission of temporal lobe seizures (19), especially in MTLE (20). Group 1 was divided into Groups 1A, B. The semiologic features of Group 1A were mainly feeling of fear, epigastric auras and oroalimentary automatisms. However, the semiologic features of Group 1B were dominated by manual and oroalimentary automatisms.

The feeling of fear is significantly associated with the amygdala, which is inseparable from the neurophysiological function of the amygdala (21–23). Epigastric auras is the most common type of MTLE and is associated with the medial temporal structure, limbic circuitry and insula cortex (24, 25). The anterior insula cortex may be the symptomatogenic zone of epigastric auras (26). We targeted temporal structure studies to correlate the occurrence of epigastric auras with the hippocampal body. Hippocampal sclerosis is the most common pathological type of MTLE (27), and the hippocampus is the predominant epileptogenic region. Substantial evidence suggests that epileptogenic networks often involve broader regions beyond the hippocampus (28). Therefore, the production of epigastric auras in MTLE may be mainly related to the spread of epileptic discharges in the hippocampal body to the anterior insula cortex.

Oroalimentary automatisms are significantly correlated with the temporal pole, hippocampal tail, and amygdala. It is a common manifestation of MTLE and is a common semiologic feature in groups 1A, B; its production in temporal lobe seizures may require a broader epileptogenic network. Jasper (29) found that stimulating the amygdala only creates feeling of fear when the after discharges are confined to the amygdala and hippocampus, and automatisms occur when the after discharges spread more widely to the temporal, orbitofrontal and central cortices. In addition, temporal pole plays a vital role in TLE (19). The temporal pole activity can mimic the semiologic features of medial temporal and frontal epilepsy (30). When temporal pole discharge spreads preferentially to the frontal lobe, it may produce hyperkinetic. Moreover, temporal pole discharges may produce loss of awareness and automatisms when it spreads preferentially to the medial temporal lobe. In a study (20), oroalimentary automatisms occurred in **20 of 23** patients with early temporal pole involvement in seizures. And in another study (30), oroalimentary automatisms occurred in 15 of the 19 patients with temporal pole epilepsy. Compared with MTLE, the first clinical sign and loss of awareness occurred sooner in the temporal pole epilepsy (20). The occurrence of oroalimentary automatisms in temporal lobe seizures may be caused by a synchronized spread of discharge from medial temporal lobe structures to the insula and operculum areas (31), especially to the anterior insula and frontal operculum (32). Our findings suggested that the abnormal discharge of structures in this group easily spread to the insular lobe. Other previous studies have also described the correlation between the amygdala or temporal pole and oroalimentary automatisms (33, 34), while in our study, the production of oroalimentary automatisms was also significantly associated with the hippocampal tail. As the main epileptogenic region of MTLE, the hippocampus is connected to the mammillary body through the fornix, which is the primary efferent fiber of the hippocampus. The hippocampal tail links the hippocampus and the fornix in this nerve conduction, so it makes sense that the hippocampal tail is significantly correlated with oroalimentary automatisms.

The semiologic features of Group 1A are similar to those of Group 1. However, the semiologic feature of Group 1B is oroalimentary automatisms, which is also a common feature of Groups 1A, B, and its production requires a relatively extensive epileptogenic network. Therefore, the structures in group 1A may be the main symptomatogenic zone or in an important position in the relevant epileptogenic network of medial temporal lobe seizures, while the structure of Group 1B may serve more the role of a “relay point” to assist the propagation of medial temporal lobe seizures to other regions.

### The Anatomic-Electroclinical Correlation of Group 2

Group 2 consisted of temporal cortical structures and correlated significantly with auditory auras, focal hypokinetics, and some elementary motor signs. Group 2 was divided into Groups 2A, B. In these subgroups, Heschl's gyrus has a closer anatomical adjoinance to the superior temporal gyrus but divides with the inferior temporal gyrus and the fusiform gyrus into a subgroup, which we considered is because during seizures, only a small number of seizures involve Heschl's gyrus, the inferior temporal gyrus and the fusiform gyrus (Table 2). These structures are often rated on a 0 point at the same time when scoring the ictal signs of the seizures. However, based on clustering results, the inferior temporal gyrus and the fusiform gyrus tend to be affected simultaneously, and their epileptogenic discharges are more easily transmitted to occipital and parietal lobes.

The primary auditory cortex in humans is located in Heschl's gyrus, with simple auditory auras closely related to it (35), while more complex auditory hallucinations may be related to the superior temporal gyrus (36, 37). Ictal signs in both Groups 2A, B were predominantly elementary motor signs. When considering the whole course of seizures, after a period of focal signs, head/eye deviation, asymmetric facial contraction, and asymmetric tonic posture usually mark the onset of GTCS (38, 39). Although may not be truly generalized (40), GTCS caused by focal seizures also involves extensive cortical and subcortical networks (41). Compared to MTLE, GTCSs are more frequent in LTLE (9). Both the middle temporal gyrus and the fusiform gyrus are part of the cortical structure of the temporal lobe. The fusiform gyrus is located at the bottom of the temporal lobe, and when the epileptiform discharge spreads to this region, perhaps its diffusion range is already relatively extensive. The middle temporal gyrus is located in the middle of the temporal cortex, and a large range of temporal cortical firing is more likely to be involved here. In the initial phase of LTLE generalization, most patients have elementary motor signs, such as head/eye deviation, unilateral versive signs, and asymmetric tonic posture. GTCS will occur when the thalamic reticular nucleus is involved. Notably, it has been suggested that more significant activation of the posterior lateral temporal regions occurs in GTCSs prior to propagation to other cortical regions (42). In our results, both Groups 2A, B were characterized by elementary motor signs. However, the activation of the superior and middle temporal gyrus was more significant than that of the inferior temporal gyrus and fusiform gyrus in LTLE.

In this study, the temporal structures were grouped in greater detail through cluster analysis, and ictal signs significantly correlated with some temporal structures were found, which could effectively assist in the localization of the EZ and guide the formulation of SEEG implantation surgery or EZ resection surgery.

## Limitations

This study had some limitations. First, it was a single-center retrospective study. Due to the limited time of SEEG recording, the semiology does not appear every time, the seizures in some patients occur during sleep, and the patient forgets after seizures, it was challenging to record auras. Second, during intracranial electrode implantation, the implanted areas had to be implanted as determined by the preoperative assessment. Therefore, it is impossible to collect discharge conditions for all brain regions. For some unsampled sites, we cannot wholly exclude involvement in the network of seizures. Third, MTLE patients occupied a more significant proportion of this cohort, with a smaller proportion of LTLE patients, so larger datasets and more detailed recordings of intracranial discharge conditions are still needed.

## Conclusion

In conclusion, temporal structures can be grouped based on the degree of involvement in seizures. Structures within each subgroup are often closer in anatomical positions than structures that are not in the same subgroup. Epilepsy is a network disorder in which clustering of anatomical structures helps us identify which structures are more likely to belong to the same network. Further correlation studies demonstrate that semiology analysis is essential in a more detailed diagnosis of anatomical localization. Presurgical localization can be helped by identifying specific semiologic patterns of temporal lobe seizures.

## Data Availability Statement

The raw data supporting the conclusions of this article will be made available by the authors, without undue reservation.

## Ethics Statement

The studies involving human participants were reviewed and approved by the Ethics Committee of Sanbo Brain Hospital, Capital Medical University. Written informed consent to participate in this study was provided by the participants' legal guardian/next of kin. Written informed consent was obtained from the individual(s), and minor(s)' legal guardian/next of kin, for the publication of any potentially identifiable images or data included in this article.

## Author Contributions

BZ, JZ, and GL designed the research. BZ and JW wrote the paper. BZ and MW performed the data analysis. ZL and PX performed the clinical data and literature search. XW, TL, YG, MZ, CL, YZ, and MZ contributed to the clinical interpretation. JZ and GL were the guarantors. All authors contributed to the article and approved the submitted version.

## Funding

This work was supported by the National Key R&D Program of China (2020YFC2007304) and National Natural Science Foundation of China (81790654).

## Conflict of Interest

The authors declare that the research was conducted in the absence of any commercial or financial relationships that could be construed as a potential conflict of interest.

## Publisher's Note

All claims expressed in this article are solely those of the authors and do not necessarily represent those of their affiliated organizations, or those of the publisher, the editors and the reviewers. Any product that may be evaluated in this article, or claim that may be made by its manufacturer, is not guaranteed or endorsed by the publisher.
